# Knowledge of psychosocial factors associated with low back pain amongst health science students: a scoping review

**DOI:** 10.1186/s12998-019-0284-5

**Published:** 2019-11-15

**Authors:** Kelsey L. Lewis, Patrick J. Battglia

**Affiliations:** Logan University Health Centers, Logan University, 1851 Schoettler Road, Chesterfield, MO 63017 USA

**Keywords:** Psychosocial factors, Low back pain, Health occupation students, Education

## Abstract

**Background:**

Low back pain is a burden worldwide and biological, psychological, and social mechanisms play a role in its development and persistence. Current guidelines support care using the biopsychosocial model. However, biomedical constructs dominate clinician training, and it is unknown the extent to which health science students understand the psychosocial determinates of a patient’s low back pain. Therefore, the aim of this scoping review is to report health science students’ current knowledge of psychosocial factors associated with low back pain.

**Methods:**

A scoping review framework was used to search electronic databases for research examining health science students’ knowledge of psychosocial factors associated with low back pain. The nature and findings of the studies are highlighted using the data charting tool. Each study was analyzed to determine the type of outcome measurement used. Scores were compared to minimum accepted scores, between disciplines, as education advanced, and after educational modules.

**Results:**

Fourteen studies published between 2004 and 2019 were identified. Seven healthcare disciplines were represented.

In total, 12 different measurement tools were utilized. In 9 studies students demonstrated inadequate knowledge of psychosocial factors associated with low back pain. Three tools compared disciplines and nationalities. Three tools were associated with practice behavior. Eight studies showed improvement as students’ education advanced, and 3 studies demonstrated improvements in knowledge after implementation of pain education modules of varied lengths. Of those, two showed significant improvement.

**Conclusions:**

Health science students in these studies had substandard understanding of psychosocial factors associated with low back pain. Dedicated pain education has the potential to improve low back pain understanding, resulting in more guideline appropriate care recommendation.

## Background

Low back pain is a costly and prevalent global health issue [1–3]. It is the leading cause of disability worldwide [3]. According to the Global Burden of Disease study in 2015, there has been a 54% increase in years lived with disability from low back pain since 1990 [4]. Often multiple and simultaneous mechanisms cause low back pain requiring a biopsychosocial perspective to optimize care [1]. Although tissue damage may have been the origin of low back pain, in the majority of cases a specific physical injury cannot be identified [5]. Numerous psychosocial factors interact with the neurobiology of ongoing low back pain, which is often refractory to monotherapy approaches [5]. Depression, distress, depressive mood and somatization are predictors of chronicity and disability in low back pain patients [5]. Psychological factors associated with chronic pain in general (i.e. not just low back pain) include depression, anxiety, post-traumatic stress disorder, fear avoidance, catastrophizing, kinesiophobia, poor coping strategies, poor self-efficacy, and pre-existing somatization [5–8]. Social factors include work absenteeism, isolation, legislation, compensation systems, and social and economic infrastructures [5, 9, 10].

There is a discrepancy between evidence-based guidelines for the management of low back pain and clinical practice [3, 11, 12]. Guidelines support multidisciplinary care for persistent low back pain including education, continuing with usual activities, exercise, manual therapy, and psychological treatments. This approach is more consistent with holistic care and the biopsychosocial model. However, care is often biomedical and fragmented with recommendations for rest and prescription medications [12]. It has been postulated that this translation gap facilitates the continued poor outcomes in low back pain management [1, 12].

Many different health care professions treat patients with low back pain, and students of these varied disciplines represent the future of pain management. Patients report that health care professionals have the strongest influence on their beliefs about LBP, thus provider beliefs dictate outcomes [13–15]. If students have adequate knowledge of psychological and social factors contributing to low back pain it is likely they will make more effective treatment recommendations that align with evidence-based guidelines. The purpose of this scoping review is to evaluate health science students’ knowledge of psychosocial factors associated with low back pain.

## Methods

Scoping review methodology as described by Arskey and O’Malley, and later revised by Levac then Tricco was utilized to collect and organize relevant information and then provide a comprehensive examination of the existing body of literature [16–18]. This is completed in 6 stages:

1. Identifying the research question
2. Identifying relevant studies
3. Study selection
4. Charting the data
5. Collating, summarizing, and reporting the results
6. Consulting with key stakeholders

We followed the recently standardized PRISMA-ScR extension by Tricco et al. (http://www.equator-network.org/reporting-guidelines/prisma-scr/) [18]. We deviated from the 6 steps proposed by Arksey and O’Malley because we have no key stakeholders to consult with. There is no mandatory scoping review registration of scoping review protocols.

Stage 1: identifying the research question

This review evaluated the question ‘do health science students have knowledge of psychosocial factors associated with low back pain?’

Stage 2: identifying relevant studies

We searched the following electronic databases: PubMed, Google Scholar, EMBASE (Ovid), SINAHL, MEDLINE, Cochrane library, Index of Chiropractic Literature, and PEDro from database inception to May 22, 2019. The ‘related articles’ features were utilized and references of all relevant papers were inspected. The search was completed using a combination of MeSH terms, keywords, and ChiroSH terms, which can be found in Appendix 1. Additionally, grey literature (e.g. conference proceedings) were searched using the same terms. Back pain belief and pain education were searched relative to the many health care disciplines and the associated student of those disciplines (physiotherapy, physical therapy, medicine, chiropractic, acupuncture, psychology, nursing, and social worker) that commonly treat low back pain.

Stage 3: study selection

All abstracts were screened independently by both authors (KL, PB). Publications identified as potential for inclusion were gathered in full-text and the same inclusion process was performed. Any disagreements on inclusion were resolved through discussion.

Inclusion criteria: participants were health science students of any discipline, and an assessment tool was utilized to measure their knowledge of psychosocial factors associated with low back pain.

Exclusion criteria: participants were not students (e.g. practitioners), no assessment tool was used to measure their knowledge of psychosocial factors associated with low back pain, or assessment of knowledge was not specific to low back pain.

Stage 4: charting the data

We classified the data based on author, year, study population, methodology, aims of the study, outcome measures, and results and conclusions. The research form was tested independently by the two authors to determine consistency, accuracy, and completeness of approach. There was no quality assessment, as there was much heterogeneity between publications and the goal was to assess the entirety of the literature on the topic.

Stage 5: collating, summarizing, and reporting the results

We analyzed the studies by determining how students were measured on their knowledge of psychosocial factors and where that measurement fell when compared to the minimum accepted score. From there we determined if students had adequate knowledge of psychosocial factors associated with low back pain.

Stage 6: considering our research question, we did not think it appropriate to consult with key stakeholders

## Results

Fourteen studies were identified [19–32], published between 2004 and 2019. Students examined include: physical therapy (PT) alone (*n* = 4), PT compared with nursing (*n* = 1), PT compared with medical and nursing (*n* = 1), PT compared with occupational therapy (OT) and nursing (*n* = 1), PT compared with non-healthcare (n = 1), medical alone (*n* = 3), medical compared to non-healthcare (n = 1), osteopathy (n = 1), and medical, chiropractic, OT, PT, and pharmacists were compared (n = 1). In total, 4964 students were studied.

The main outcomes of each study are presented in Table 1. The following assessment tools were used: Healthcare Providers’ Pain and Impairment Relationship Scale (HC-PAIRS) (*n* = 9); Back Pain Beliefs Questionnaire (BBQ) (*n* = 3); Questionnaire assessing likely practice behaviors (*n* = 1); 21-questions assess clinical judgement (*n* = 1); Interpersonal Reactivity Index (IRI): Empathy components (*n* = 1); Fear Avoidance Beliefs Questionnaire- Physical Activity component (FABQ-PA) (*n* = 1); an objective structured clinical examination (OSCE) (*n* = 1); Modified Back Pain Beliefs Questionnaire (MBBQ) (*n* = 1); Back Pain Attitudes Questionnaire (Back-PAQ) (*n* = 1); Pain Attitudes and Beliefs Scale (PABS) (n = 1); and the Modified Photograph Series of Daily Activities (MPHODA) (*n* = 1).

**Table 1:** Data charting tool for included studies

Author [Year of publication]	Study population	Methods	Aims of the study	Outcome measures	Results and conclusions
Ferreira PH et al. [2004] [19]	3rd and 4th year Brazilian physiotherapy students (*n* = 153)	• Survey study design utilizing the HC-PAIRS which was translated from English to Portugese. • Student in their 3rd and 4th years had already a taken a musculoskeletal course, but had not treated nor observed the management of patients with LBP. The results of the survey were compared to data published by Latimer et al. (2004) which included 3rd and 4th year physiotherapy from Australia (*n* = 618). The scores of the Brazilian and Australian students were compared with scores of healthcare providers reported by Rainville et al. (1995).	• Compare HC-PAIRS of Brazilian students with Australian students. • Compare students’ scores to those of North American Healthcare Providers.	HC-PAIRS^a^	• All HC-PAIRS scores were higher than the recommended 38. • Brazilian students had significantly higher total HC-PAIRS scores than Australian students. • Brazilian students had higher scores on every factor of the HC-PAIRS with the greatest differences being seen in factors 1 and 3. • Factor 4 scored the lowest. • They conclude Brazilian students agree more strongly that pain justifies limitation in function, and that ethnicity can influence beliefs about low back pain.
Latimer J et al. [2004] [20]	3rd (98′, 99′, 00′) & 4th (98′) year physiotherapy students (*n* = 618)	• Survey administered pre- and post- 16 h course on chronic low back pain. • The 98′ and 00′ 3rd year students were surveyed immediately before and after the module. • The 99′ class was surveyed before and one year after taking the module.	To describe the attitudes and beliefs of physiotherapy students to chronic LBP and investigate whether these attitudes changed following exposure to a teaching module.	HC-PAIRS	• All HC-PAIRS scores were higher than the recommended 38, indicating stronger beliefs that pain justifies disability and functional limitation. • In the 98′ cohort there was improvement in factors 1 and 2. • In the 00′ cohort the improvement was seen in factors 1 and 2. • Factor 4 did not improve significantly in either group. The 99′ cohort that was tested 1 year after taking the module had significant improvement in total scores and factors 1–3. • They conclude that attitudes and beliefs of 3rd year physiotherapy students can be changed following a teaching module, and that these changes are still presents one year later. However, these scores are still above the maximum recommended score of 38.
Burnett A et al. [2009] [21]	2nd-4th year undergraduate physiotherapy and nursing students from Australia, Taiwan, and Singapore (*n* = 382).	Cross-sectional survey study	To determine if country (Australia, Taiwan, and Singapore), undergraduate healthcare major (physiotherapy and nursing), LBP history, and year of course influenced back pain beliefs in undergraduate females.	• HC-PAIRS • BBQ^b^	• All HC-PAIRS scores were higher than the recommended 38. • HC-PAIRS scores were lower in physiotherapy students compared to nursing students. • No significant difference between HC-PAIRS scores of physiotherapy students from Australia, Singapore, and Taiwan. • 3rd year Australian physiotherapy students had significantly lower HC-PAIRS scores than 2nd year. • Amongst nursing students HC-PAIRS scores were lowest in Taiwanese students, followed by Australian and Singaporean. • BBQ scores were significantly lower in nursing students versus PT students. • BBQ scores were significantly lower in Taiwanese and Singaporean students as compared to Australian students. • There was a significant correlation between BBQ and HC-PAIRS scores. • They conclude that Chinese students have more negative beliefs about functional limitations associated with low back pain as compared to white Australian students.
Ryan C et al. [2010] [22]	1st & 4th year physiotherapy (*n* = 62) & non-healthcare students (n = 61)	Cross-sectional survey study	To investigate the difference in attitudes between 1st and 4th year physiotherapy students and between physiotherapy students and non-healthcare students toward functioning in individuals with LBP.	HC-PAIRS	• All HC-PAIRS scores were higher than the recommended 38. • First year physiotherapy students had lower total and factor 1 scores than non-healthcare students. • Fourth year physiotherapy students had lower total scores and all factors except 4 when compared with non-healthcare students. • There was a greater gap between 4th year physiotherapy students and non-healthcare students than 1st year physiotherapy students and non-healthcare. • Fourth year physiotherapy students scored lower than 1st year students in total and on all 4 factors except factor 4. • There was more improvement in HC-PAIRS scores in physiotherapy students than non-healthcare students, however there scores were still greater than 38. • They conclude physiotherapy education creates more positive attitudes toward the functioning of individuals with low back pain than non-healthcare education.
Morris H et al. [2012] [23]	1st (n = 62) & final year (n = 61) medical students & business students as controls	Cross-sectional survey study	To investigate whether medical student training fosters positive attitudes towards patients with LBP and their ability to function.	HC-PAIRS	• All HC-PAIRS scores were higher than the recommended 38. • Undergraduate medical students had lower HC-PAIRS scores than business students, and the scores decreased between 1st and final year students. • There was no improvement in factor 3, but factors 1 and 2 improved in final year students. • They conclude that final year medical students develop appropriate attitude toward people with chronic low back pain, but that more education is needed because the scores did not reach the recommended 38.
Briggs AM et al. [2013] [24]	Final year students (*n* = 602): medicine (*n* = 176), chiropractic (*n* = 46), occupational therapy (*n* = 71), pharmacy (*n* = 138), & physiotherapy (*n* = 171)	Cross-sectional survey study	To investigate beliefs and clinical recommendations for LBP, and their alignment to the current evidence-based guidelines, in Australian university allied health and medical students.	• HC-PAIRS • BBQ • Questionnaire to assess likely practice behavior^c^	• All HC-PAIRS scores were higher than the recommended 38. • Physiotherapy and chiropractic students had lowest HC-PAIRS scores (better), and pharmacy students had the highest. • The HC-PAIRS scores were strongly associated with clinical recommendations. • Physiotherapy and chiropractic students scored higher on the BBQ, while pharmacy students reported the lowest scores. • BBQ scores were associated with clinical recommendations as well. A 1-pt increase in BBQ was associated with an increase in the odds of recommendations consistent with guidelines with the exception of work recommendations. • Physiotherapy and chiropractic students demonstrated more “guideline consistent” recommendations as far as physical activity, work, and bedrest were concerned. • Pharmacy and occupational therapy students were least likely to recommend consistent with the guidelines. • They conclude that across disciplines, physiotherapy and chiropractic students have more helpful beliefs about functioning with low back pain than occupational therapy, pharmacy, or medical students. They believe this is a reflection of the curricula for physiotherapy and chiropractic students having more low back pain related modules.
Chinball JT et al. [2014] [25]	Final year medical students (*n* = 132)	• Randomized experiment using 4 vignettes were patients with LBP were seeking long-term disability. • Levels of patient accountability were manipulated by having the patient only be seen 1 time (weak) or for long-term care (strong). • Levels of societal accountability were manipulation by having the student be the secondary source for disability award (weak) or the primary (strong). • Clinical judgements (symptom validity, pain/distress/disability (PDD), and psychosocial factors) and empathy were measured.	• Investigate the effects of empathy and accountability (patient and societal) on clinical judgements of medical students relative to patients and legal/societal obligations.	• Questionnaire with 21 clinical judgment questions^d^ • 7 Empathetic concern items from the IRI^e^	• The mean empathy score was 19.5 indicating slightly greater than moderate levels of self-reported empathy. • Higher empathy scores were associated with higher symptom validity and P/D/D, but not with psychosocial factors. • There was no clear relationship between high patient or societal accountability alone and clinical judgements. • When patient and societal accountability were consistent (ie. both weak or strong) symptoms were viewed as less valid, and P/D/D and psychosocial factors played a greater role. • When patient and societal accountability were inconsistent (ie. 1 weak, 1 strong) symptoms were viewed as more valid and P/D/D and psychosocial factors played less of a role. • When societal accountability was weak symptoms were viewed as more valid in those who had high patient accountability. Psychosocial factors and PDD were considered low. • When Societal accountability was high and students’ patient accountability was low (ie. 1-time consultation) symptom validity was low and P/D/D and psychosocial factors were high. • They suggest that students take the path of least resistance when seeing a patient only 1 time, and validate their symptoms, thus awarding their disability. OR students who anticipate ongoing care believe psychosocial factors are a malleable component of the pain experience and they will direct their treatment toward those.
Kennedy N et al. [2014] [26]	Physiotherapy (*n* = 107), medicine (*n* = 63), nursing (including midwifery) (*n* = 101) students	Cross-sectional survey administered to physiotherapy, medical, and nursing students of all years.	To compare the belief of Irish university physiotherapy, medical, and nursing students toward LBP and to investigate whether demographics (current or previous LBP, gender, and year of study) influence these beliefs.	• BBQ • FABQ-PA^f^	• Physiotherapy students had significantly higher BBQ and lower FABQ-PA scores than medical and nursing students. • Medical students had significantly higher BBQ scores than nursing students. • Beliefs among physiotherapy and medical student improved as their education advanced (1st vs. 4th year students). • BBQ and FABQ-PA scores improved to indicate more positive beliefs as year of study progressed in both physiotherapy and medical students. • Physiotherapy students have more positive beliefs toward LBP than medical and nursing students. Physiotherapy and medical students beliefs significantly improved over the course of their studies.
Weiner DK et al. [2014] [27]	3rd year medical students not exposed to pain module (*n* = 28), and those exposed to the module (*n* = 27)	Cross-sectional survey study	To describe development of an educational module on evaluation and treatment of chronic LBP in older adults and the effect of the module on medical students clinical skills.	OSCE^g^	• Following implementation pain module students were assessed via an OSCE. • The components of the OSCE included history and physical examination. • 96% of students enrolled in the low back pain module passed the OSCE, compared with 61% of students not enrolled who passed. This was a statistically significant improvement (*P* < .001) • They conclude that the findings are encouraging about the potential for educational interventions to positively affect evaluation and management of chronic low back pain in adults.
Alshami AM et al. [2015] [28]	3rd or 4th year physiotherapy students from Saudi Arabia (*n* = 135) compared with data published by Latimer et al. (2004) and Ferreira et al. (2004)	Cross-sectional survey study administered to Saudi Arabian students. Those scores were compared to previously reported scores on Brazilian and Australian students. The HC-PAIRS was translated to Arabic.	To investigate the attitudes and belief of Saudi Arabian physical therapy students toward chronic LBP. Scores were compared to previously published data on Brazilian and Australian students.	HC-PAIRS	• All HC-PAIRS scores were higher than the recommended 38. • Saudi Arabian students had significantly higher total HC-PAIRS scores, and higher scores in factors 1 and 2. • There was no difference between scores in 3rd and 4th year physiotherapy Saudi Arabian students. • All groups had poor factor 4 scores. • They conclude there are differences in beliefs about functioning with low back pain between different nationalities, and that physiotherapy students at junior levels had more negative beliefs about low back pain.
Abdel Shaheed C et al. [2017] [29]	First year medical students (*n* = 93)	Cross-sectional survey using the MBBQ that was administered to medical students pre- and post-15-min educational video.	To explored medical students’ knowledge, attitudes, and beliefs toward LBP before and after a 25-min educational video on LBP.	• MBBQ^h^	• Following the educational intervention students demonstrated improvement in the inevitability score, and the proportion of students who answered correctly on items dealing with activity, bed rest, imaging, and recovery. • Post-intervention students’ knowledge, attitudes, and beliefs toward LBP improved and aligned more closely with current evidence-based guidelines. • Authors conclude that educational interventions do not need to be extensive in order to improve students’ knowledge, attitudes, and beliefs regarding LBP.
Hilbink H [2017] [30]	New Zealand osteopathy students (*n* = 83) in all years of their education. Year 1 (*n* = 12) Year 2 (*n* = 17) Year 3 (*n* = 21) Year 4 (*n* = 14) Year 5 (*n* = 19)	Online cross-sectional survey administration.	To identify common LBP attitudes and beliefs of New Zealand osteopathy students.	• Modified HC-PAIRS • Back-PAQ^i^	• The median Back-PAQ score was 6.5. The score increased with more education (year 1 vs. year 4). • Students had the most negative scores in ‘the need to protect’ theme, and highest scores in ‘the correlation between pain and injury’. • The median HC-PAIRS score was 46. Scores decreased as education advanced. • Osteopathy have less than optimal attitudes and beliefs about the back and back pain that are not consistent with current guidelines. However, improvements were seen as education advanced.
Leysen M et al. [2017] [31]	2nd (*n* = 766) and 4th (*n* = 584) year physiotherapy students in 6 different Universities in Belgium.	Cross-sectional survey.	• To explore attitudes and beliefs of bachelor and master physiotherapy students in Belgium toward LBP.	• PABS^j^ • HC-PAIRS • Vignette with associated questionnaire to assess treatment recommendations ^k^	• 2nd year students scored significantly higher on the PABS- BM scale and on the HC-PAIRS. • 4th year students scored significantly higher on the PABS-PS sub-scale. • After reading the vignette, 4th year students had more correct (guideline-consistent) answers regarding activity and work. However, only 45% of 4th year students answered correctly (compared with 15% of the 2nd year students). • The PABS-BM was negatively correlated with PABS-PS and positively correlated with the HC-PAIRS. • The HC-PAIRS correlated negatively with the PABS-PS. • 4th year students have more biopsychosocial beliefs and attitudes about LBP compared with 2nd year students. However, guideline-consistent recommendations is low in all students.
Leahy A et al. [2019] [32]	Physiotherapy (*n* = 115), Occupational therapy (OT) (*n* = 48), and general nursing (*n* = 79) students at any point in their education at the University of Limerick in Ireland.	Cross-sectional survey of physiotherapy, occupational therapy, and general nursing pre-registration students in Ireland.	To investigate the beliefs of healthcare students about how harmful common daily activities are perceived to be for their lower back.	• MPHODA survey^jl^	• Physiotherapy students had significantly more positive beliefs where they considered activities of daily living to be less harmful for the lower back followed by occupational therapy, and lastly nursing students. • Physiotherapy students later in their education had more positive beliefs than year 1 students, but the same was not true for OT and nursing students.

^a^The Healthcare Providers’ Pain and Impairment Relationship Scale (HC-PAIRS) consists of 15 items with each item rated on a Likert scale from 1 (completely disagree) to 7 (completely agree). Scores range from 15 to 105, and higher scores indicate stronger beliefs that low back pain justifies disability and limited activity. The modified version consists of 13 items and replaces “chronic low back pain” with “low back pain”. The scoring remains the same. The tool has 4 sub scales (with corresponding numbers of the HC-PAIRS):

1. functional expectations

2. social expectations

3. need for a cure

4. projected cognition

^b^The Back Pain Beliefs Questionnaire (BBQ) consists of 14 statements on a 5-point Likert scale ranging from 1 (completely disagree) to 5 (completely agree), and scores range from 9 to 45. Lower scores represent negative beliefs about future consequences of a life with low back pain. These consequences include illness progression, disability, and effectiveness of treatments

^c^The questionnaire to assess likely practice behavior consists of 3 items rated on a 5-point Likert scale investigating health professional’s recommendations for patient behavior in relation to work, activity, and bed rest. Lower scores represent more restrictive recommendations. The questionnaire is used following a vignette describing a 28 year-old woman with acute non-specific low back pain without red flags. Responses to the vignette are considered either ‘guideline consistent’ or ‘guideline inconsistent’

^d^21-questions assessing clinical judgement rated on a Likert scale of 0–10. Higher scores indicate greater amounts of the variable being measured (e.g. Patient honesty in presentation. 0 = not at all, 10 = definitely)

^e^Interpersonal Reactivity Index (IRI): Empathy components (n = 7) are rated on a 0–4 Likert scale with total scores ranging from 0 to 28. Higher scores indicate higher levels of empathy. The full IRI includes other sub-scales (Fantasy, involvement, personal distress, and perspective talking) that were not used in the study

^f^Fear Avoidance Beliefs Questionnaire (FABQ) consists of 2 sub-scales for physical activity (PA) and work (W). The FABQ-PA was used to determine the level of fear-avoidant behaviors that healthcare students display in their everyday lives. Four individual items are scored on a Likert scale of 0–6. The total score ranges from 0 to 24 with lower scores indicating more positive beliefs about physical activity and higher scores indicating more fear-avoidant beliefs about physical activity

^g^Objective structure clinical examination (OSCE) is a versatile multipurpose clinical evaluation tool used to assess a number of clinical competencies through objective observation. The OSCE utilized in Weiner et al.’s study included standardized patients with chronic low back and leg pain. The students were asked to take a history, perform the physical examination, or do the documentation. The faculty directly observing the student marker a “yes” or “no” if the students performed a particular task. Students were given real-time feedback

^h^Modified Back Pain Beliefs Questionnaire (MBBQ) that asks students to rate their agreement with statements on a 1–5 Likert scale (1 = strongly disagree, 5 = strongly agree). There are 25 items, wherein the first 14 were taken from the BBQ, and items 15–25 were sourced from the 11-item Buchbinder scale. Preferred responses are based on evidence-based management of low back pain. The “inevitability score” was calculated based on 9 inevitability items of the questionnaire comprised of questions regarding physical activity, bed rest, return to work, and recovery within the context of acute LBP. A lower inevitability score is considered more favorable, and a mean score change of 3 is considered to be a meaningful effect

^i^Back Pain Attitudes Questionnaire (BACK-PAQ) uses 34 items to evaluate students attitudes and beliefs about their own back and back pain, rather than their beliefs about their patients’ back pain. Each item is rated on a 5-point Likert scale (− 2 to + 2; “false” to “true”). Scoring ranges from − 68 to 68, and although there is no cut off for “good” or “bad” attitudes and beliefs, more negative scores indicate negative beliefs, and positive scores indicate positive beliefs that are in line with current guidelines. The internal consistency is rated using Cronbach’s alpha of 0.70–0.91. There are 6 themes within the questionnaire:

1. The vulnerability of the back

2. The need to protect the back

3. The correlation between pain and injury

4. The special nature of back pain

5. Activity participation while experiencing back pain

6. The prognosis of the back

^j^Pain Attitudes and Beliefs Scale (PABS) contains 2 sub-scales: the biomedical (BM) and psychosocial (PS). The questionnaire is used to assess students affinity toward either a biomedical model or a biopsychosocial model when it comes to the management of LBP. It is comprised of 19 items: 10 biomedical and 9 behavioral. Each item is scored on a 1–6 Likert scale (1 = Totally disagree and 6 = Totally agree). Higher scores in the different sub-scales indicate a stronger affinity for that model

^k^Vignette with associated questionnaire to assess treatment recommendations. No additional information can be ascertained about this questionnaire because this resource was an abstract presented at a conference

^l^Modified Photograph Series of Daily Activities (MPHODA) survey which was derived from the Photograph Series of Daily Activities survey comprised of 100 pictures. The MPHODA consists of 12 pictures indicative of various postures that load the spine in day-to-day activities. Students rate each picture on a Likert scale of 0–100 where 0 = not harmful and 100 = extremely harmful. Lower scores indicate more positive beliefs

In 9 studies students failed to demonstrate adequate knowledge of psychosocial factors associated with low back pain. With respect to the HC-PAIRS, students consistently scored higher than the recommended 38, indicating more negative beliefs about disability and functional limitations due to low back pain [33]. The BBQ was used to compare disciplines and showed physical therapy consistently scored higher than medical, occupational therapy, pharmacy, and nursing students. Chiropractic students scored higher than occupational therapy, pharmacy, and nursing students in 1 study [24]. Higher scores indicate more positive beliefs about the future in patients with LBP [34]. The FABQ-PA and MPHODA were also used to compare disciplines. The former found that PT students have less fear-avoidant beliefs than medical and nursing students [26]. The latter demonstrated that PT students had significantly more positive beliefs where they considered activities of daily living to be less harmful for the low back than OT and nursing students [32]. The questionnaire to assess future practice behavior developed by Evans et al. (2005) was used to compare disciplines [35]. Chiropractic and physical therapy students were more likely to make ‘guideline consistent’ recommendations as compared to occupational therapy and pharmacy students. These recommendations correlated with HC-PAIRS and BBQ scores. Utilizing the Back-PAQ and HC-PAIRS, Hilbink determined that osteopathy students have less than optimal knowledge of psychosocial factors and beliefs about LBP that are not consistent with guidelines [30].

Chibnall et al. reported that students are more likely to recognize psychosocial factors at play when they have an ongoing relationship with the patient rather than a 1-time evaluation. [[25] In this study, students were seeing patients that were seeking disability. Students were either seeing the patients long-term (strong patient accountability), or 1-time (weak patient accountability). Additionally, the student was either the primary doctor being used to grant the patient disability (strong societal accountability) or they were the secondary doctor being seen (weak societal accountability). When patient and societal accountability were consistent (ie. both strong or both weak) students viewed psychosocial factors as playing a major role in the pain presentation. This was demonstrated using 4 clinical vignettes followed by the IRI-empathy components and the questionnaire with 21-items assessing clinical judgement [25].

Several studies demonstrated improvements in scores on various outcome measures as education advanced [20–23, 26, 30–32].

Several studies implemented educational modules ranging from 15-min to 16-h [20, 27, 29]. They all determined that knowledge of psychosocial factors and beliefs about LBP improve following the educational interventions. However, the HC-PAIRS scores were still sub-optimal in 1 study [20]. Weiner et al. utilized an OSCE and Abdel Shaheed et al. utilized the MBBQ to evaluate students pre- and post-educational intervention. Both demonstrated significant improvements. The former with a significant amount of students passing the LBP OSCE, and the latter with more students answering with positive beliefs and guideline-consistent answers on the MBBQ [27, 29].

## Discussion

This scoping review highlights certain health science students’ inadequate knowledge of psychosocial factors contributing to the development and persistence of low back pain regardless of discipline. Differences were seen between disciplines and nationalities. Students’ knowledge of psychosocial factors seems to improve as their education advances, although still to a sub-optimal level in most. Dedicated pain education has potential to improve knowledge.

The leading cause of years lived with disability in both developed and undeveloped countries is low back pain, and 40% of adults report living with low back pain [3, 36]. A 2006 review found that the total cost of low back pain exceeds $100 billion yearly, and that two-thirds of these costs are due to lost wages and reduced productivity [37]. An impact of that magnitude necessitates better understanding of the multidimensional nature of low back pain to provide more precise and cost-effective care.

The biopsychosocial model describes the inseparable interaction between biological (somatic), psychological, and social contributors to a person’s health [38, 39]. This is exemplified in low back pain as over 90% of diagnoses are non-specific, indicating a poor correlation between physical findings and pain [2]. Current guidelines for the management of non-specific low back pain support a biopsychosocial framework [1, 2, 11]. Our review demonstrates students’ general lack of knowledge of psychosocial factors associated with low back pain. For low back pain treatment, there is a gap between evidence and practice, suggesting first-line treatments like promoting activity and function, including work participation are not being readily recommended [12]. For example, Rainville et al. found that the mean HC-PAIRS score of orthopedic surgeons and family physicians in the U.S. was 54 (i.e. higher than the recommended 38), and that this score was associated with inappropriate practice recommendations endorsing activity restrictions and rest [40]. Based on our results, more training is needed at the health science student level to improve their understanding of psychosocial factors associated with low back pain. Deliberate training on this topic may improve future practice recommendations.

In this review there was heterogeneity in evaluating students’ knowledge. Several different measurement tools were utilized, none of which comprehensively measured the students’ knowledge of psychological and social factors associated with low back pain. Several authors suggest removing factor 4 (projected cognition) from the HC-PAIRS after noting the poor correlation between factor 4 and the remaining factors, and thus questioning factor 4’s psychometric value [19, 22, 24]. As for the BBQ, FABQ, MBBQ, Back-PAQ, PABS, and MPHODA there are no set standard for the scoring system, but rather higher or lower scores indicating more negative or positive beliefs when compared across disciplines. A systematic review found that OSCEs are a valid way to test students, and they’re advantageous for evaluating real-time clinical decision-making [27, 41].

Despite the lack of uniformity in assessing students, some conclusions can be made. Students from most health care disciplines lack satisfactory knowledge of psychosocial factors associated with low back pain. In a small sample, physical therapy and chiropractic students scored significantly better on the HC-PAIRS than occupational therapy, medical, and pharmacy students. Those same students were more likely to make ‘guideline consistent’ recommendations later in practice. Using the PABS-BM/PS, Leysen et al. discovered that 4th year PT students have more biopsychosocial beliefs than 2nd year, and that they’re more likely to answer correctly with guideline-consistent responses. However, even in this study the correct responses were made by only 45% of 4th year PT students. In general, physical therapy students demonstrate more positive beliefs about low back pain as compared to nursing, occupational therapy, and medical students. Differences in nationality were also associated with different HC-PAIRS scores. In general, Australian students had more positive beliefs, although still sub-optimal, as compared to Brazilian, Singaporean, Taiwanese, and Saudi Arabian students. As students progress in their education they have improved HC-PAIRS, FABQ-PA, Back-PAQ, and PABS scores, indicating health care education improves knowledge of psychosocial factors associated with low back pain. However, in the majority of the studies showing improvements the authors’ conclude that students still need continuing education on guideline-consistent LBP care because the improvement seen was not significant. There was improvement following dedicated pain modules seen in Latimer et al., Weiner et al., and Abdel Shaheed et al. [20, 27, 29] The first reported improved scores to still be sub-optimal; the second indicated significant improvement in the number of students who passed the chronic low back pain OSCE; and the last reported improvements in knowledge, attitudes, and beliefs toward LBP and greater alignment with evidence-based guidelines. The OSCE included history and physical exam. There may be benefit of pain-specific courses highlighting chronic pain and treatment which included addressing psychosocial factors. Future studies should determine the appropriate length and mode of teaching a pain course.

Interest in students understanding of the biopsychosocial model relating to pain has grown recently. A recent review of 20 studies evaluating students knowledge, skills, attitudes, or beliefs about pain across medicine, nursing, PT, and occupational therapy found that students lack these key components to adequately manage pain and many had negative attitudes and beliefs about pain [42].

Ultimately, care adhering to the biopsychosocial model should yield better outcomes [39]. Psychological interventions decrease disability and pain catastrophizing, and improve activity levels, pain control, and illness perceptions in patients with low back pain [43, 44]. A recent qualitative systematic review determining the effect of psychological interventions delivered by non-psychologists (e.g. physical and occupational therapists, and physicians) on low back pain and disability found significantly decreased low back pain and disability scores. However, when compared to guideline-informed usual care the psychological interventions were superior in only a few of the studies [45]. A study conducted at the Ford Motor plant discovered that through the use of mind-body practices (e.g. yoga and tai-chi) and acupuncture there was a 58% reduction in prescription medication for low back pain management [46]. Because this research is still in its infancy more work is needed to determine its true impact. However, psychosocial factors contribute to the development and persistence of low back pain, and targeted intervention may help relieve low back pain and suffering [5].

## Limitations

Most students studied were physical therapy students. Because of this, the findings hold truer for this discipline as compared to disciplines that were not studied (e.g. clinical psychologists and surgical residents). This is an avenue for future research to evaluate all health science students’ knowledge of psychosocial factors associated with low back pain.

## Conclusions

Students across many healthcare disciplines do not have adequate knowledge of psychosocial factors associated with low back pain. Therefore, it is likely that suboptimal low back pain care will persist. Future research should determine the most effective way to implement pain courses in the curricula of health science students and evaluate the outcomes of these pain courses as they relate to improvement in student understanding and ultimately outcomes in low back pain care.

## Data Availability

Data sharing is not applicable to this article as no datasets were generated or analyzed during the current study.

## Appendix

### Electronic database search strategy

1. Psychosocial AND back AND students
2. Psychosocial AND back AND education
3. Attitudes and beliefs AND education
4. Back AND healthcare student belief
5. Back pain belief AND
6. Physiotherapy
7. Physiotherapy student
8. Physical therapy
9. Physical therapy student
10. Medical doctor
11. Medical student
12. Chiropractor
13. Chiropractic student
14. Acupuncture
15. Psychologist
16. Psychology student
17. Pain psychologist
18. Pain psychology student
19. Nursing
20. Nursing student
21. Social working
22. Student beliefs about back pain
23. Back pain attitude AND student
24. Pain education AND
25. Physiotherapy student
26. Physical therapy student
27. Medical student
28. Chiropractic student
29. Psychology student
30. Nursing student
31. Social worker
32. Back pain education AND
33. Student
34. Physiotherapy student
35. Physical therapy student
36. Medical student
37. Chiropractic student
38. Psychology student
39. Nursing student
40. Social worker

## Abbreviations

Back-PAQ: Back Pain Attitudes Questionnaire
BBQ: Back pain beliefs questionnaire
FABQ: Fear Avoidance Beliefs Questionnaire
FABQ-PA: Fear Avoidance Beliefs Questionnaire- Physical activity
HC-PAIRS: Health care providers’ pain and impairment relationship scale
IRI: Interpersonal Reactivity Index
MBBQ: Modified Back Pain Beliefs Questionnaire
MPHODA: Modified Photograph Series of Daily Activities
OSCE: Objective structured clinical examination
OT: Occupational therapy
PABS: Pain Attitudes and Beliefs Scale
PT: Physical therapy
