# Correction to: Knowledge of psychosocial factors associated with low back pain amongst health science students: a scoping review

**DOI:** 10.1186/s12998-019-0289-0

**Published:** 2019-11-26

**Authors:** Kelsey L. Lewis, Patrick J. Battaglia

**Affiliations:** Logan University Health Centers, Logan University, 1851 Schoettler Road, Chesterfield, MO 63017 USA

**Correction to: Chiropr Man Ther (2019) 27:64**

**https://doi.org/10.1186/s12998-019-0284-5**

Following publication of the original article [1], we have been notified that one of the author names was incorrect.

Originally published author name:

- Patrick Battglia

Correct author name:

- Patrick Battaglia

The original article has been corrected.
